# Supplementary material for: Septoria Leaf Blotch and Reduced Nitrogen Availability Alter WRKY Transcription Factor Expression in a Codependent Manner
Source: Int J Mol Sci. 2020 Jun 11;21(11):4165. doi: 10.3390/ijms21114165 (PMC7312603; doi:10.3390/ijms21114165)
Supplement: Supplementary file 1 [file ijms-21-04165-s001.pdf]

Supplementary Tables

Supplementary Table 1 Primers sequences to amplify sections of transcripts in wheat or regions in *Zymoseptoria tritici*. Non-standard base codes are R = A or G, Y = C or T, M = A or C.

Target Gene	Forward Primers Sequence	Reverse Primer Sequence
**Internal Transcribed Spacer (ITS)**	GGAAGTAAAAGTCGTAACAAGG	TCCTCCGCTTATTGATATGC
**Ubiquitin**	ACCATTGACAACGTGAAGGC	TGGATGTTGTAGTCCGCCAAG
**WRKY2**	CTCTTTGGCTTCTCCTTTCACG	TGTTGTTGTTGCTGCTGCTG
**WRKY10**	AATTTCGAAGCCGGTGATCC	CCGTACATGTTCATCGTCTCG
**WRKY19**	TTGGCAACTTCAGTGCTGAC	ACCAGTGTGTGATGGCAAAG
**WRKY53b**	GCCATGTCCTCCTCCACG	ACAGAAGCTCGGTGAAGGAC
**WRKY68a**	GACCATGGCCGTGGACCC	CGGAGAGGGAGGAGACGAG
**WRKY39**	AATTTAAGAAAGACATGGACGAGCA	CACGAGGATCTTGGTCACCG

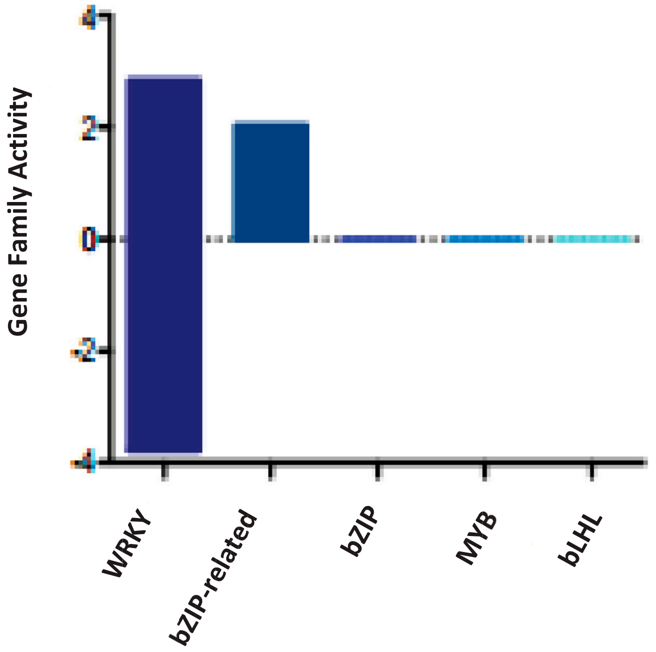

Supplementary Figure 1. Variation in expression of transcription factor superfamily genes in wheat under reduced nitrogen conditions in the field analysed by microarray. WRKYs show clear up- and down- regulation in response to nitrogen stress, whilst bZIP-related genes are upregulated. Differential expression of bZIP, MYB and bHLH transcription factor families is not seen in response to low nitrogen.

20

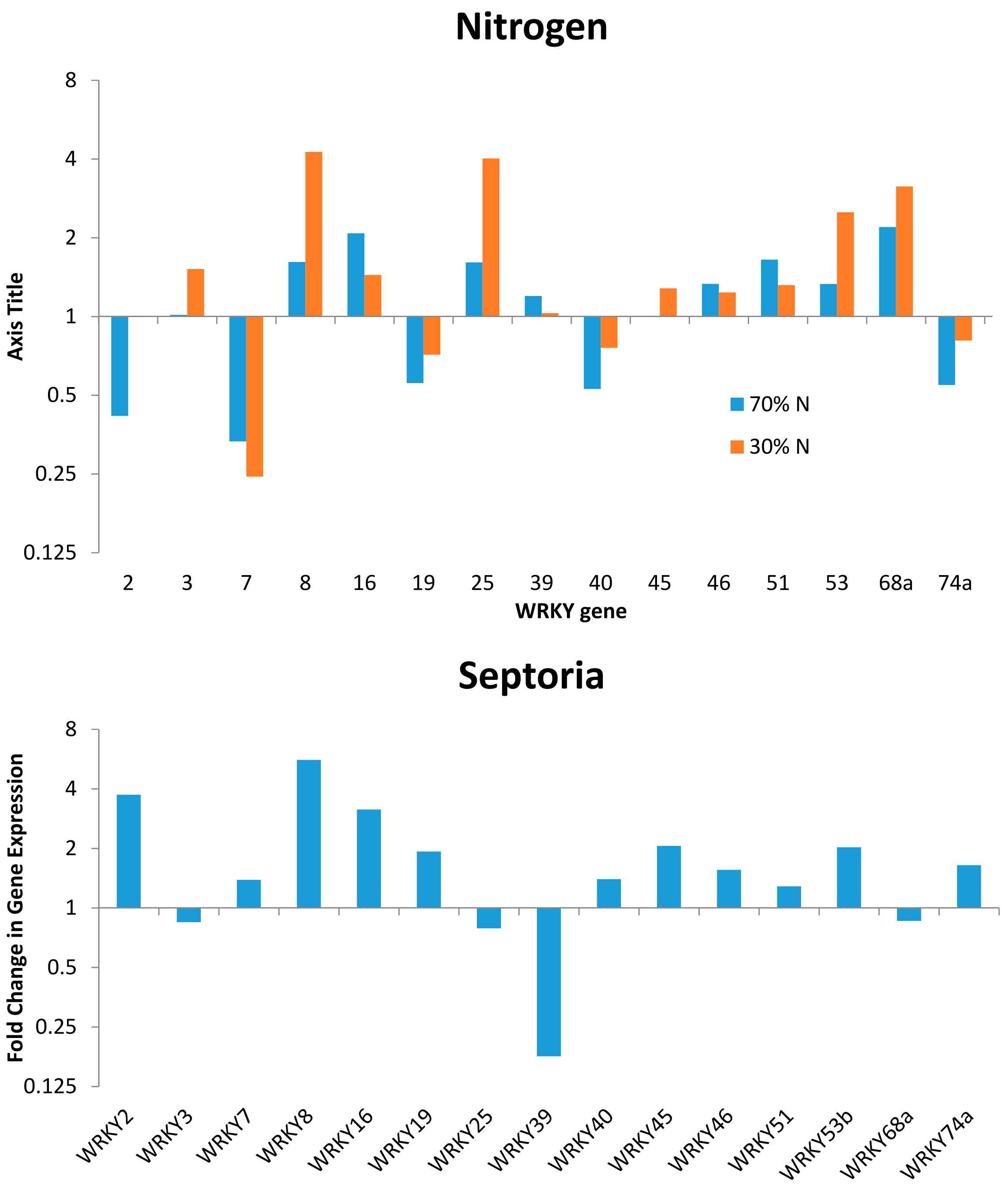

Supplementary Figure 2. Changes in expression of WRKYs for which coding sequences were publically available at the time and for which transcripts were detectable in our samples in response to nitrogen reduction and *Septoria* infection. Whilst some WRKYs, such as WRKY8, demonstrated promising results in response to both stressors, sequence similarity to other WRKYs meant it was not possible to obtain a single PCR product, and thus qPCR results were deemed to unreliable to continue including in the analysis.

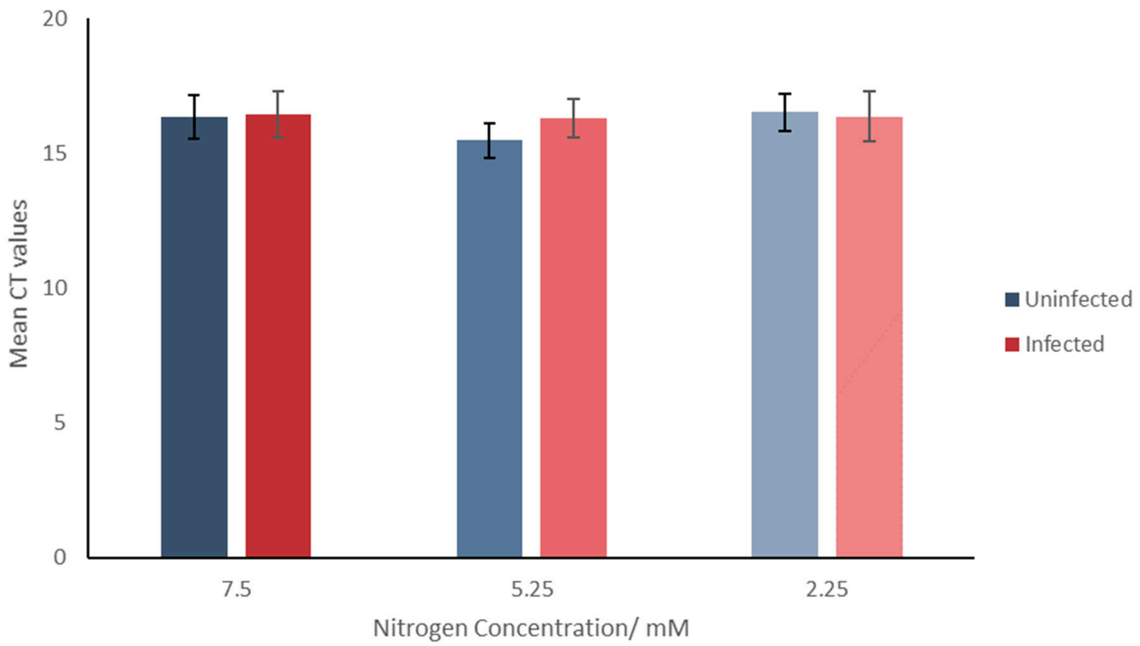

Supplementary Figure 3 Expression of *EF1α* was consistent across nitrogen concentrations and infection status with no significant treatment effect seen between the six conditions (p = 0.478). This confirmed the suitability of *EF1α* as an endogenous control to enable normalisation against total amount of cDNA. Means compared using one-way ANOVA. n = 3 for uninfected conditions, n=5 for infected conditions.
